# Utility of ISARIC 4C Mortality Score, Vaccination History, and Anti-S Antibody Titre in Predicting Risk of Severe COVID-19

**DOI:** 10.3390/v16101604

**Published:** 2024-10-12

**Authors:** Lin Pin Koh, Travis Ren Teen Chia, Samuel Sherng Young Wang, Jean-Marc Chavatte, Robert Hawkins, Yonghan Ting, Jordan Zheng Ting Sim, Wen Xiang Chen, Kelvin Bryan Tan, Cher Heng Tan, David Chien Lye, Barnaby E. Young

**Affiliations:** 1 National Centre for Infectious Diseases, Singapore 308442, Singapore; kohlinpin@gmail.com (L.P.K.); travischia@hotmail.com (T.R.T.C.); samuel.wang@mohh.com.sg (S.S.Y.W.); jean-marc_chavatte@moh.gov.sg (J.-M.C.); cherhengtan@ntu.edu.sg (C.H.T.); david_lye@ncid.sg (D.C.L.); 2 Lee Kong Chian School of Medicine, Nanyang Technological University, Singapore 308232, Singapore; 3 Department of Infectious Diseases, Tan Tock Seng Hospital, Singapore 308433, Singapore; 4 Department of Laboratory Medicine, Tan Tock Seng Hospital, Singapore 308433, Singapore; robert_hawkins@ttsh.com.sg; 5 Department of Radiology, Tan Tock Seng Hospital, Singapore 308433, Singapore; yonghan_ting@nuhs.edu.sg (Y.T.); jordansim92@gmail.com (J.Z.T.S.); chen_wen_xiang@ttsh.com.sg (W.X.C.); 6 Ministry of Health, 16 College Road, College of Medicine Building, Singapore 169854, Singapore; kelvin_bryan_tan@moh.gov.sg; 7 Yong Loo Lin School of Medicine, National University of Singapore, Singapore 117597, Singapore

**Keywords:** COVID-19, SARS-CoV-2, serology, modelling, vaccination, ISARIC

## Abstract

The ISARIC 4C Mortality score was developed to predict mortality risk among patients with COVID-19. Its performance among vaccinated individuals is understudied. This is a retrospective study of all patients with SARS-CoV-2 infection admitted to the National Centre for Infectious Diseases, Singapore, from January-2020 to December-2021. Demographic, clinical, and laboratory data were extracted, and multiple logistic regression (MLR) models were developed to predict the relationship between ISARIC score, vaccination status, anti-S antibody titre, and severe COVID-19. A total of 6377 patients were identified, of which 5329 met the study eligibility criteria. The median age of the patients was 47 years (IQR 35–71), 1264 (23.7%) were female, and 1239 (25.7%) were vaccinated. Severe disease occurred in 499 (9.4%) patients, including 133 (2.5%) deaths. After stratification, 3.0% of patients with low (0–4), 17.8% of patients with moderate (5–9), and 36.2% of patients with high (≥10) ISARIC scores developed severe COVID-19. Vaccination was associated with a reduced risk of progression to severe COVID-19 in the MLR model: aOR 0.88 (95% CI: 0.86–0.90), and the risk of severe COVID-19 decreased inversely to anti-S antibody titres. The anti-S antibody titre should be further investigated as an adjunct to the ISARIC score to triage COVID-19 patients for hospital admission and antiviral therapy.

## 1. Introduction

Severe acute respiratory syndrome coronavirus 2 (SARS-CoV-2), the causative agent of coronavirus disease 2019 (COVID-19), was first reported in Wuhan in December 2019 [1]. The virus spread globally, and in the first two years of the pandemic alone, it has been estimated that 3.4 billion cases occurred, with 18.2 million attributable excess deaths [2,3].

Hospitalisations due to COVID-19 have put a significant strain on healthcare systems worldwide and continue to do so during waves of infection associated with the emergence of new variants [4]. Prognostic scores assist healthcare providers to rapidly triage patients by identifying who is at the highest risk of severe illness and, therefore, should be prioritised for hospitalisation and antiviral treatment. One of the most robust examples was developed relatively early during the pandemic, in 2020, and is known as the International Severe Acute Respiratory and Emerging Infectious Consortium (ISARIC) 4C (Comprehensive Clinical Characterisation Collaboration) Mortality score [5,6,7].

However, this score was developed based on patient data collected early during the pandemic, before SARS-CoV-2 vaccines became available and when the vast majority of the population was seronegative. With the vast majority of the global population now vaccinated and many multiply infected, further study on the performance of the ISARIC 4C Mortality score is needed to assess its continued utility. There are also limited data on whether serological markers such as the spike (anti-S) antibody titre can be used to predict a patient’s risk of severe COVID-19. This has become increasingly relevant as vaccine uptake declines, the interval between SARS-CoV-2 infections extends, and, thus, antibody levels wane. Quantitative anti-S data could also guide determining in which individuals oral antiviral therapy is cost-effective.

We conducted a retrospective cohort study of all patients hospitalised with COVID-19 at the National Centre for Infectious Diseases (NCID), Singapore, from January 2020 to December 2021, with the aim of studying the utility of the ISARIC 4C Mortality score, vaccination history, and the anti-S antibody titre in predicting the risk of severe COVID-19. This dataset is unique, as, during this period, anti-S antibody titres were routinely measured as part of standard clinical management, and many patients were admitted to hospital for public health purposes rather than clinical-care needs.

## 2. Materials and Methods

### 2.1. Data Collection and Ethics Statements

We conducted a retrospective cohort study with a waiver of informed consent approved by the institutional ethics committee (National Healthcare Group Domain Specific Review Board reference number 2020/01122). All patients with SARS-CoV-2 infection confirmed by polymerase chain reaction (PCR) and admitted to the NCID from January 2020 to December 2021 were included. Patients’ demographic, clinical, and laboratory information were extracted from the hospital medical databases. Radiographic evidence of COVID-19 pneumonia was determined using a previously published artificial intelligence programme [8].

### 2.2. Pandemic Response Measures in Singapore

On 23 January 2020, Singapore reported its first confirmed case of COVID-19 [9]. Extensive contact tracing for all confirmed cases was initiated with testing and mandatory isolation of cases and exposed contacts under the Infectious Diseases Act. Following a large community outbreak of the Delta variant from April 2021 and with 67% of the population fully vaccinated, these measures were gradually relaxed from 6 August 2021 [10]. From 15 September 2021, a home recovery programme was implemented, and hospitalisation was prioritised for individuals identified as either being at high risk for progression to severe COVID-19 or requiring inpatient care for clinical reasons [11].

Singapore launched its COVID-19 National Vaccination Programme (NVP) on 30 December 2020 with the Pfizer–BioNTech/Comirnaty vaccine (BNT162b2) [12]. The Moderna (mRNA-1273) mRNA vaccine was approved for use under the NVP on 17 March 2021 [12]. The initial vaccination strategy prioritised individuals at high risk for severe COVID-19 and/or SARS-CoV-2 infection such as older adults and frontline healthcare workers, respectively.

### 2.3. Predictors for Severe COVID-19

The ISARIC 4C Mortality score at admission was retrospectively calculated for each patient using available data. Patient age, sex, comorbidities (using the non-age-adjusted Charlson Comorbidity Index [CCI] as a proxy), serum urea, and C-reactive protein (CRP) were available [6,7]. However, respiratory rate (RR), peripheral oxygen saturation, and Glasgow Coma Scale (GCS) were omitted from the calculations as these data could not be extracted electronically. When included for comparison purposes, ISARIC 4C Mortality scores were categorised into low (0 to 4), moderate (5 to 9), or high (≥10).

The number of vaccine doses each patient had received at the time of index-COVID-19-related hospital admission was recorded. Patients were stratified into vaccinated (those with two or more vaccine doses at the time of infection) or unvaccinated (those with one or zero vaccine doses at the time of infection).

For the serology analysis, we extracted SARS-CoV-2 anti-spike (anti-S) antibody titres measured using the Roche Elecsys^®^ immunoassay. These were routinely measured at admission as part of clinical care from 29 September 2020.

### 2.4. Outcomes

The primary study outcome was progression to severe COVID-19 within 28 days of admission. Severe COVID-19 was defined as requirement for supplemental oxygen during hospitalisation, admission to the intensive care unit, and/or death. All patients with severe illness on admission were excluded from further analysis.

### 2.5. Statistical Methods

All statistical analyses were performed using R-Studio (2022.07.01+554). Multiple logistic regression (MLR) was used to determine relationships between predefined predictors and outcomes. Additionally, we conducted separate analyses for each component variable after first calculating the score based on the ISARIC 4C Mortality score formula.

## 3. Results

**Figure 1:**
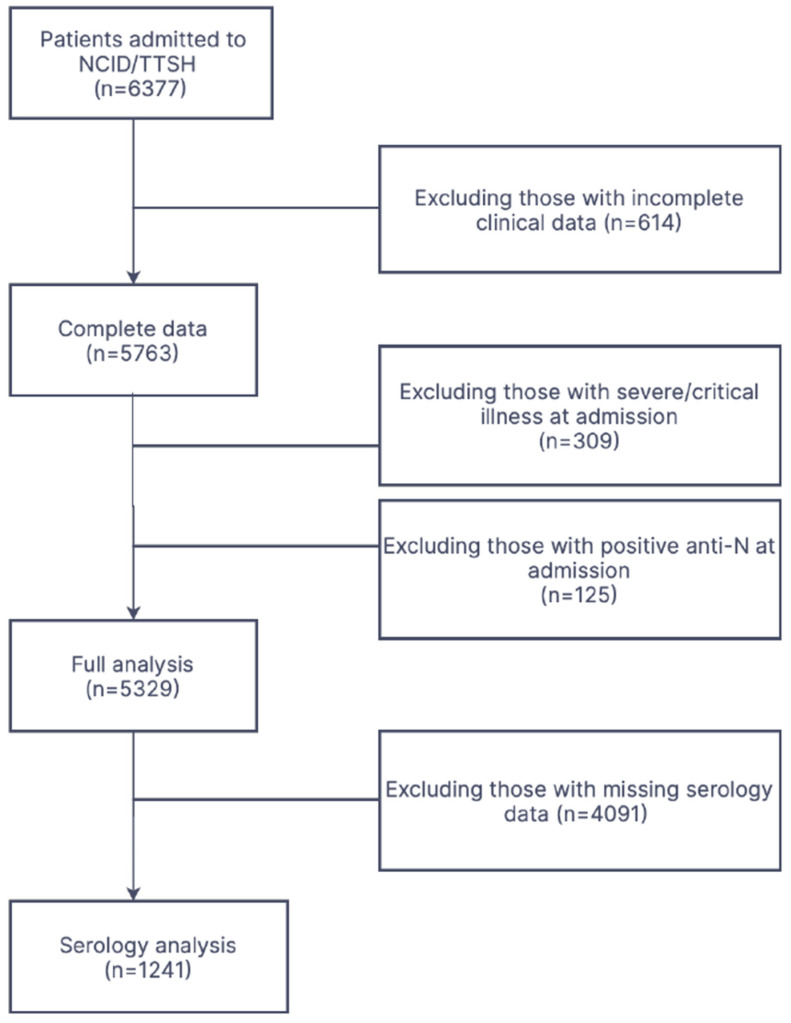
Study flow diagram.

During the study period, 6377 patients were admitted to the NCID with SARS-CoV-2 infection, and 5329 met the study eligibility criteria (Figure 1). Individuals with positive anti-N serology were assumed to have prior infection and were thus excluded to reduce the possibility of previous infections, which could be a confounding factor in immunity and disease outcomes [13]. The median age of this group was 47 (interquartile range [IQR] 35–71) years, and 1264 (23.7%) were female. The median CCI was 0 (IQR 0–0), and 1239 (25.7%) were vaccinated. Severe COVID-19 occurred in 499 (9.36%) patients, including 133 (2.50%) deaths, 96 (1.80%) ICU admissions, and 409 (7.67%) who required supplemental oxygen.

**Figure 2:**
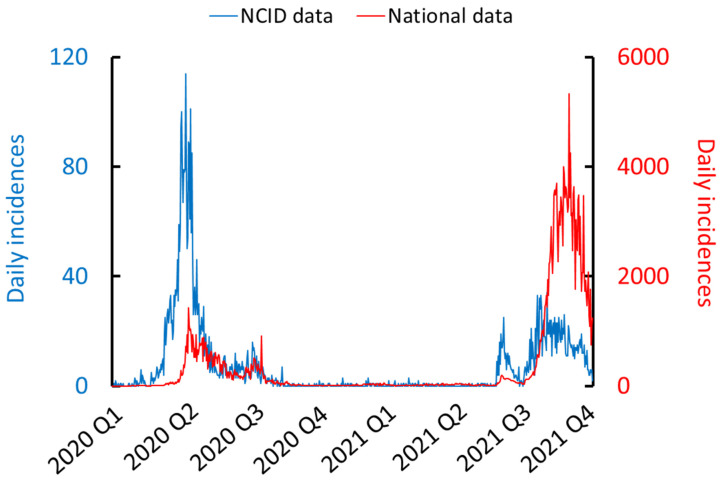
COVID-19 daily incidences for the full analytical dataset of 5329 subjects admitted to the NCID (blue) and that for total daily incidences in Singapore based on WHO data (red). A total of 7.0% of patients in the first wave (2020 Q1 and Q2) were included in our analytical dataset while only 1.0% of remaining patients (2020 Q3 to 2021 Q4) were in the same dataset.

During the ‘ancestral’ COVID-19 wave from January to September 2020, there were 3393 patients, with a median age of 39 (IQR 32–50) years, and 425 (12.5%) were female. Our dataset includes approximately 7.0% of all COVID-19 diagnoses in Singapore during this period, when a larger proportion of cases were admitted to hospital for isolation (Figure 2). October 2020 to April 2021 corresponds with the emergence of the Alpha and Beta variants of concern. Containment measures effectively limited the number of cases in Singapore over this period to relatively few. Finally, from May 2021 to 1 December 2021, the emergence of the Delta variant resulted in significant community transmission. Reflecting changes in the national containment strategy, during this period, the median age of patients was 74 (IQR 60–83) years, and 807 (43.2%) were female. The baseline characteristics of the study cohort are summarised in Table 1.

**Figure 3:**
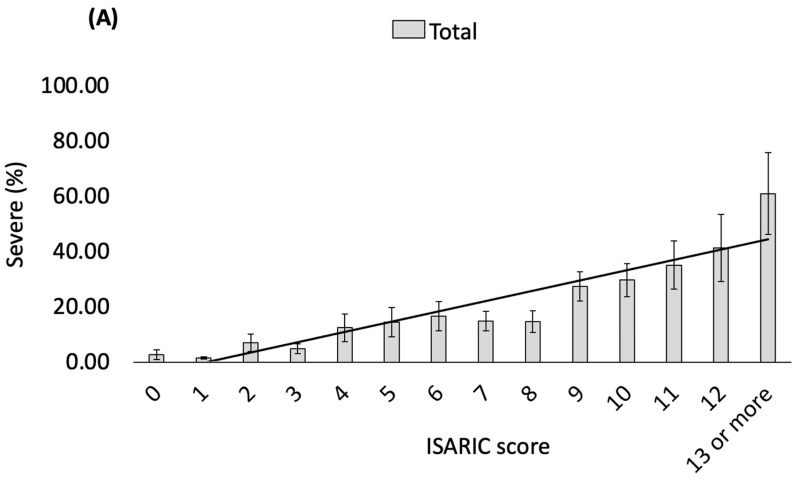

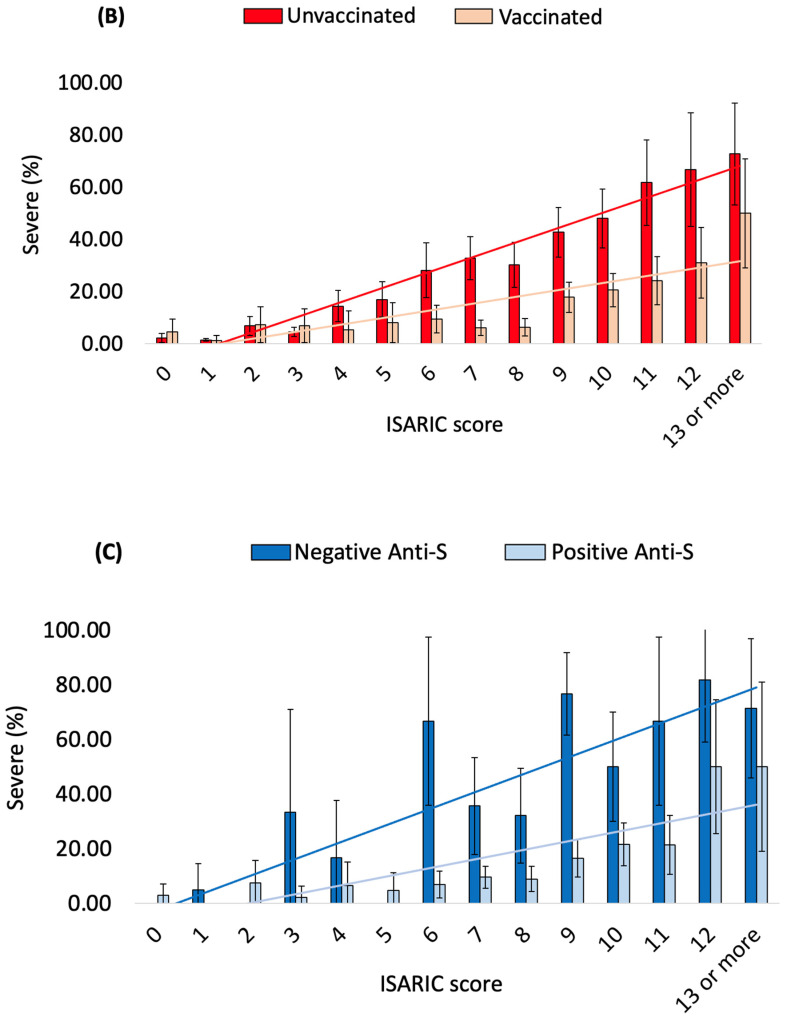
(**A**) Percentage of severe COVID-19 at each ISARIC 4C score in the whole cohort (*n* = 5329). (**B**) Percentage of severe COVID-19 among unvaccinated and vaccinated patients at each ISARIC 4C score (*n* = 5329). Risk of severe COVID-19 > 20% at ISARIC 4C of 6 for unvaccinated and ISARIC 4C of 9 for vaccinated patients. (**C**) Percentage of severe COVID-19 among patients with negative and positive anti-S antibody (*n* = 1241). Risk of severe COVID-19 > 20% at ISARIC 4C of 3 for negative anti-S antibody and ISARIC 4C of 10 for positive anti-S antibody. Error bars represent 95% confidence intervals.

Analysing the full dataset of 5329 patients, all available components of the ISARIC 4C Mortality score, apart from male sex, were associated with progression to severe disease in the multivariable analysis as expected (adjusted odds ratio (aOR) > 1): age aOR: 1.02 (95% CI: 1.02–1.03), sex aOR: 0.98 (95% CI: 0.96–0.99), comorbidities aOR: 1.04 (95% CI: 1.03–1.05), serum urea aOR: 1.09 (95% CI: 1.07–1.10), CRP aOR: 1.13 (95% CI: 1.11–1.15) (see Supplementary Table S1). Vaccination was associated with a reduced risk of severe COVID-19: aOR 0.88 (95% CI: 0.86–0.90). This was also observed after stratifying patients based on the ISARIC 4C Mortality Score (Figure 3).

**Figure 4:**
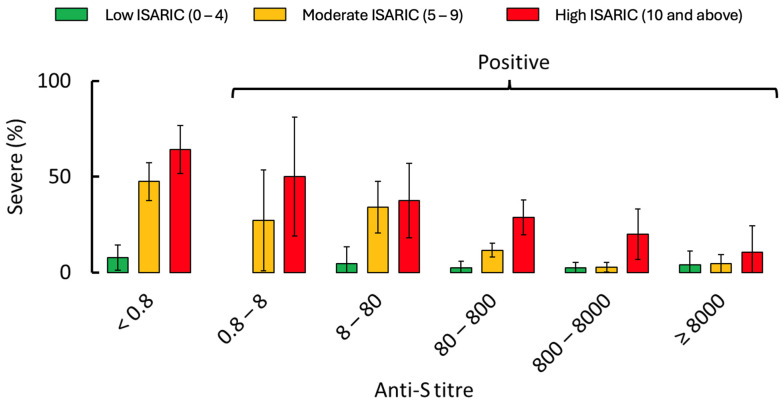
Percentage of patients with progression to severe COVID-19 in the serology dataset (*n* = 1241) is affected by anti-S titre when stratified by ISARIC. Error bars represent 95% confidence intervals.

In the subset of patients with serology results (*n* = 1241), 904 (87.3%) of nonsevere COVID-19 cases had positive anti-S serology compared with 117 (57.1%) of severe COVID-19 cases (Table 1). When stratified by the ISARIC 4C Mortality score, the proportion of people with negative anti-S who developed severe COVID-19 rose from 7.7% among those with a low ISARIC score to 64.3% for those with a high ISARIC score (Supplementary Table S2). Among those with positive anti-S titre, the risk of severe COVID-19 was significantly lower, and the proportion of severe COVID-19 rose from 2.6% at a low ISARIC score to 27.5% at a high ISARIC score. Furthermore, positive anti-S status strongly correlated with a reduced proportion of severe illness in the multivariate model (aOR: 0.83; 95% CI: 0.78–0.88), as did a history of vaccination (aOR: 0.92; 95% CI: 0.87–0.97) (Table 2). Both the vaccination and the serology status correlated with a lower risk of severe COVID-19 when the ISARIC 4C was low but a higher risk at moderate or high ISARIC 4C scores (Figure 3 and Figure 4, and Supplementary Table S1).

The effect of vaccination status and anti-S antibody titre on disease progression was similar. A ≥20% risk of severe COVID-19 was observed in vaccinated patients with an ISARIC 4C Mortality score ≥ 9, but among unvaccinated patients, this risk was observed as a score of ≥6 (Figure 3B). Similarly, for patients with a negative anti-S antibody titre, a ≥20% risk of severe COVID-19 was observed when the ISARIC 4C Mortality score was ≥3, but among patients with a positive anti-S titre, a risk of ≥20% was only observed when the score was ≥10 (Figure 3C). At a very low ISARIC 4C Mortality score, the anti-S antibody titre did not modify the risk of severe COVID-19 (Figure 4). For a moderate ISARIC 4C Mortality score, the risk of severe COVID-19 decreased as the anti-S antibody titres increased up to 800 U/mL, and a very low risk of severe COVID-19 was observed when the anti-S antibody titre was >800 U/mL. However, for patients with a high ISARIC 4C Mortality score, the increased risk of severe COVID-10 persisted even as the anti-S antibody titre exceeded 8000 U/mL.

## 4. Discussion

Early during the COVID-19 pandemic, many healthcare systems worldwide faced significant strain due to the overwhelming volume of cases [4]. Accurate predictors of disease severity can assist healthcare providers by identifying which patients require hospital admission and/or early therapeutics. However, as pandemics evolve, new variants emerge, and population immunity increases, it is important to re-evaluate diagnostic and therapeutic strategies.

For COVID-19, the emergence of variants from Alpha to Omicron, as well as successful vaccination programmes, have contributed to the dramatic reshaping of the pandemic. In our cohort, vaccination was a strong predictor of protection against severe COVID-19, and a history of vaccination added additional data to the predictive value of the ISARIC 4C Mortality score for predicting progression. As would be expected, vaccination had a bigger absolute impact in reducing severe COVID-19 in patients with high ISARIC 4C Mortality scores (≥10) from 59.5% to 24.5% and for those with low ISARIC 4C Mortality scores (0–4) (from 4.5% to 3.0%). The relative reduction in progression to severe illness was similar. We reported similar findings from the analysis of a smaller cohort of patients admitted during the Delta outbreak in October 2021 [14].

Since the emergence of Omicron, vaccination status has been of limited practical use as a predictor of progression. This is due to the high level of population immunity, with the majority of the global population being both vaccinated and infected. More complex assessments are required to predict how vaccination and infection history modify the risk of progression to severe illness. This may include the number of vaccine doses a patient has received, the number of infections experienced, the time since the last vaccination or infection, and whether an antigenically distinct strain has recently emerged. Although some recent studies [15] have shown that anti-S serology is not predictive of infection for Omicron, there is a lack of studies looking at severe illness. In the current COVID-19 landscape, where SARS-CoV-2 is considered an endemic respiratory virus infection, it makes sense to focus our efforts on identifying risk factors for severe illness rather than infection. Furthermore, given that multiple studies have shown that vaccines are effective at preventing severe illness in Omicron [16], it is likely that anti-S serology will be predictive of protection against hospitalisation.

Like qualitative vaccination/infection status, a qualitative anti-S antibody titre (i.e., positive or negative) offers limited discriminative potential. Outside of very young children, most of the world’s population is expected to be anti-S seropositive. However, our study suggests that a quantitative anti-S titre may be useful. While anti-S titres are typically measured in a laboratory setting, commercial assays such as the Roche Elecsys^®^ kit (Roche Diagnostics, Indianapolis, IN, USA) evaluated here are relatively simple and straightforward to perform. Point-of-care quantitative SARS-CoV-2 anti-S assays have also been developed [17,18]. The analysis presented here requires validation in a larger dataset and in the Omicron era, but we observed that the anti-S titre added clinically meaningful predictive information, particularly when estimating the risk of severe COVID-19 among patients with high ISARIC 4C Mortality scores. For patients with a moderate ISARIC 4C score, the risk of severe COVID-19 was much reduced if their anti-S antibody titre on admission exceeded 800 U/mL. For patients with a high ISARIC 4C score, the risk of severe COVID-19 declined with increasing anti-S antibody titre, though it remained significant even if the anti-S antibody titre was >8000 U/mL. A previous smaller study has also shown higher disease severity to be associated with lower anti-S antibody titres, but the association was not statistically significant, perhaps due to a smaller sample size [13]. The cost-effectiveness of oral antivirals such as nirmatrelvir-ritonavir in the general population has been questioned [19]. While there is a lack of data to guide clinical interpretation, measuring anti-S antibody titres to determine clinical care may be useful.

One limitation of this study is that, due to the prevailing isolation policy, our dataset was biased towards a higher proportion of cases diagnosed in Singapore earlier in the pandemic, before the emergence of the Alpha variant (63.7% were ‘ancestral’ COVID). Further work will be needed to confirm the relevance of these data to current and future SARS-CoV-2 outbreaks: no individuals in our cohort received >2 vaccine doses, and none were infected with the Omicron variant. We chose to analyse based on the ISARIC 4C Mortality score rather than the deterioration score. This was chosen based on clinical practice in Singapore at the time and the simplicity of its calculation. These scores were developed on the same dataset and yielded very high estimated risks, reflecting the emergence of a novel pathogen in a naïve population. Our interest was primarily in comparing the scores calculated rather than in studying the estimated risks, and we further simplified the scores by grouping them into low, moderate, and high risk. This was also the reason why we did not consider adding other factors such as coagulation factors (D dimer) or the hematologic index. However, a future study including these factors might lead to an improvement in the predictive ability of the models. Additionally, a larger dataset may have allowed for a more detailed analysis and yielded different results. This is especially the case for some ISARIC 4C Mortality scores for which our sample size is very small. Finally, we could not include some components of the ISARIC 4C Mortality score due to a lack of data. This included the Glasgow Coma Scale, respiratory rate, and peripheral oxygen saturation on room air. However, all individuals with severe COVID-19 at the point of hospitalisation were excluded from further analysis.

It is also important to recognise the limitations of using anti-S antibody titres as a risk marker for severe COVID-19. Antibody levels can wane over time, especially several months after vaccination or natural infection, potentially reducing their predictive value [20].

Lastly, the bulk of our cohort comes from the first wave of COVID-19, before the emergence of variants such as Delta or Omicron. Although this does not invalidate our findings, it does limit their applicability to the later stages of the pandemic, particularly as new variants and increasing population immunity have changed the outcomes of the disease significantly.

## 5. Conclusions

Low anti-S antibody titres are a risk factor for progression to severe COVID-19. Further work is needed to investigate whether anti-S titres could be integrated into the ISARIC 4C score and used to guide clinical care, including decisions for early antiviral therapy and hospital admission [21,22].

## Figures and Tables

**Table 1:** Comparison of demographic, vaccination, ISARIC 4C Mortality score, anti-S antibody titre and variant data among non-severe and severe COVID-19 patients.

	Non-Severe COVID (*n* = 4830)	Severe COVID (*n* = 499)	Statistical Test (Pearson’s Chi-Squared Test Unless Otherwise Indicated)
**Demographics**			
Age (IQR, years)	45 (34–68)	75 (62–85)	^ *p*-value < 0.001 ***
Sex (Male)	3768 (78.0%)	297 (59.5%)	*p*-value < 0.001 ***
**Clinical information**			
Fully vaccinated ≥ 2 doses	1239 (25.7%)	165 (33.1%)	*p*-value < 0.001 ***
ISARIC 4C (IQR)	1 (1–5)	8 (5–10)	^ *p*-value < 0.001 ***
Low ISARIC 4C 0–4	3484 (72.1%)	108 (21.8%)	*p*-value < 0.001 ***
Moderate ISARIC 4C 5–9	1064 (22.1%)	231 (46.3%)	
High ISARIC 4C ≥ 10	282 (5.8%)	160 (31.9%)	
Serum urea (±SD, mmol/L)	4.11 ± 2.74	7.25 ± 5.48	# *p*-value < 0.001 ***
C-reactive protein (±SD, mg/L)	14.94 ± 28.24	46.17 ± 50.42	# *p*-value < 0.001 ***
Lymphocyte (±SD, 10^9^/L)	1.60 ± 0.75	1.15 ± 0.63	# *p*-value < 0.001 ***
Non-age adjusted Charlson’s comorbidity index (IQR)	0 (0–0)	1 (0–2)	^ *p*-value < 0.001 ***
Pneumonia on chest X-ray on admission	720 (14.9%)	193 (38.7%)	*p*-value < 0.001 ***
Anti-S antibody status (positive)	904 (87.3%) (*n* = 1036)	117 (57.1%) (*n* = 205)	*p*-value < 0.001 ***
Anti-S antibody titre (IQR, U/mL)	454 (122–1580)	26.2 (0.8–250)	^ *p*-value < 0.001 ***
**COVID-19 presumptive variant**			
Ancestral (January–September 2020)	3230 (66.9%)	163 (32.7%)	*p*-value < 0.001 ***
Alpha/Beta (October 2020–April 2021)	53 (1.1%)	15 (3.0%)	
Delta (May–December 2021)	1547 (32.0%)	321 (64.3%)	

Results shown represent number (proportion of total *n* of column; %), mean ± standard deviation for dichotomous variables, or median (interquartile range [IQR]) for continuous variables. *** Comparison between non-severe and severe COVID-19 was statistically significant with *p* < 0.001 for all variables. # Welch’s two sample *t*-test. ^ Mann–Whitney Wilcoxon test.

**Table 2:** Univariate and multivariate models for risk of severe COVID-19 in serology cohort (*n* = 1241).

Models		Crude and Adjusted Odds Ratio with 95% Confidence Interval	
		Univariate Model	Multivariate Model
Severe COVID-19	Age (years)	1.03 (1.02–1.04) ***	1.02 (1.01–1.03) ***
	Sex (male)	0.98 (0.94–1.02)	1.00 (0.96–1.03)
	Non-age-adjusted Charlson’s Comorbidity Index	1.10 (1.07–1.12) ***	1.02 (1.00–1.05) *
	Serum urea (mmol/L)	1.19 (1.16–1.22) ***	1.11 (1.08–1.15) ***
	C-reactive protein (mg/L)	1.21 (1.16–1.26) ***	1.15 (1.11–1.20) ***
	Fully vaccinated status	0.83 (0.79–0.86) ***	0.92 (0.87–0.97) **
	Positive anti-S antibody	0.75 (0.71–0.79) ***	0.83 (0.78–0.88) ***

ISARIC 4C score components were evaluated using their score based on the ISARIC 4C formula (crude and adjusted odds ratios were calculated per increment of 1 score). Multivariate model used components of ISARIC 4C score, vaccination status, and anti-S antibody status. Serology dataset (*n* = 1241). * *p* < 0.05, ** *p* < 0.01 *** *p* < 0.001.

## Data Availability

The data supporting the findings of this study are available in the manuscript. Further data requests can be made via contact with the corresponding author.

## Supplementary Materials

The following supporting information can be downloaded at: https://www.mdpi.com/article/10.3390/v16101604/s1, Table S1: Univariate and multivariate models for risk of severe COVID-19 in full cohort (*n* = 5329); Table S2: Risk of severe COVID-19 modified by vaccination and anti-S antibody status, stratified into ISARIC 4C score categories.
