# Supplementary material for: Utility of ISARIC 4C Mortality Score, Vaccination History, and Anti-S Antibody Titre in Predicting Risk of Severe COVID-19
Source: Viruses. 2024 Oct 12;16(10):1604. doi: 10.3390/v16101604 (PMC11512353; doi:10.3390/v16101604)
Supplement: Supplementary file 1 [file viruses-16-01604-s001.zip › viruses-3201011-supplementary.pdf]

Supplementary Table S1. Univariate and multivariate models for risk of severe COVID-19 in full cohort (n=5329).

		Crude and adjusted odds ratio and 95% confidence interval	
		**Univariate model**	**Multivariate model**
**Severe COVID-19**	Age (years)	1.03 (1.03 – 1.04) ***	1.03 (1.02 – 1.03) ***
	Sex (male)	0.92 (0.90 – 0.93) ***	0.98 (0.96 – 0.99) **
	Non age-adjusted Charlson's comorbidity index	1.11 (1.10 – 1.13) ***	1.04 (1.03 – 1.05) ***
	Serum urea (mmol/L)	1.19 (1.17 – 1.20) ***	1.09 (1.07 – 1.10) ***
	C-reactive protein (mg/L)	1.19 (1.17 – 1.21) ***	1.13 (1.11 – 1.15) ***
	Fully vaccinated status	1.03 (1.02 – 1.05) ***	0.88 (0.86 – 0.90) ***

ISARIC 4C score components were evaluated using their score based on the ISARIC 4C formula (crude and adjusted odds ratios were calculated per increment of 1 score). Multivariate model uses components of ISARIC 4C score and vaccination status. **p* < 0.05, ***p*<0.01 ****p*<0.001

Supplementary Table S2. Risk of severe COVID-19 modified by vaccination and anti-S antibody status, stratified into ISARIC 4C score categories.

	Low ISARIC 0-4	Moderate ISARIC 5-9	High ISARIC 10 and above
Not/Partial vaccinated	4.5	36.5	59.5
Fully vaccinated	3.0	9.7	24.5
Negative anti-S	7.7	47.5	64.3
Positive anti-S	2.6	10.5	27.5
	Severe (%)		
